# Genetic Variants Associated With Nonpulmonary Vein Triggers of Atrial Fibrillation: A Genome-Wide Association Study

**DOI:** 10.1016/j.jacadv.2026.102839

**Published:** 2026-05-29

**Authors:** Sho Okamura, Motoki Furutani, Mika Nakashima, Noboru Oda, Takehito Tokuyama, Yousaku Okubo, Shunsuke Miyauchi, Shogo Miyamoto, Naoto Oguri, Takumi Sakai, Naoki Ishibashi, Junji Maeda, Shunsuke Ishida, Shumpei Niida, Kouichi Ozaki, Daichi Shigemizu, Hidenori Ochi, Yukiko Nakano

**Affiliations:** a Department of Cardiovascular Medicine, Graduate School of Biomedical and Health Sciences, Hiroshima University, Japan; b Medical Genome Center, Research Institute, National Center for Geriatrics and Gerontology, Aichi, Japan; c Research Institute, National Center for Geriatrics and Gerontology, Aichi, Japan; d RIKEN Center for Integrative Medical Sciences, Yokohama, Japan; e Department of Aging Research, Nagoya University Graduate School of Medicine, Nagoya, Japan; f Department of Health Management, Hiroshima Red Cross Hospital & Atomic-bomb Survivors Hospital, Japan; g Department of Gastroenterology and Metabolism, Biomedical Sciences, Graduate School of Biomedical and Health Sciences, Hiroshima University, Japan

**Keywords:** atrial fibrillation, genetic variant, nonpulmonary vein foci

## Abstract

**Background:**

Atrial fibrillation (AF) originating from non–pulmonary vein (non-PV) foci is a major cause of postablation recurrence; however, its underlying mechanisms remain poorly understood.

**Objectives:**

The purpose of this study was to identify genetic variants and electrophysiological features associated with AF originating from non-PV foci.

**Methods:**

We performed a genome-wide association study in 1,091 patients with paroxysmal AF undergoing first catheter ablation. AF triggers were classified into PV or non-PV groups based on standardized electrophysiological testing with isoproterenol provocation. AF originating from the posterior wall of the left atrium, including the PV antrum, was categorized into the PV group. Associations between genetic variants and non-PV AF triggers were assessed and validated in an independent cohort (n = 371).

**Results:**

Non-PV AF triggers were documented in 126 of 1,010 patients (12.5%) in the discovery cohort and 37 of 346 patients (10.7%) in the replication cohort. The variant rs117203318 (T>C) showed the strongest association with non-PV AF triggers (*P* = 4.65 × 10^−14^; OR: 1.98; 95% CI: 1.49-2.62). This association was replicated under Firth’s penalized logistic regression adjusted for age and sex (OR: 3.31; 95% CI: 1.40-7.55; *P* = 0.007). Non-PV AF was associated with significantly longer intra-atrial conduction times, although rs117203318 showed no correlation with conduction parameters.

**Conclusions:**

The rs117203318 variant, near genes associated with myocardial fibrosis and cellular stress responses, is associated with AF originating from non-PV foci. These findings suggest that there may be distinct substrates for non-PV AF and this could inform strategies to improve ablation outcomes.

Atrial fibrillation (AF) is the most common cardiac arrhythmia in adults that affects approximately 59 million people worldwide, including both symptomatic and asymptomatic cases.^1^ Its prevalence increases with age, and the number of patients is estimated to increase in an aging society.^2^ Paroxysmal AF (PAF) contributes to the occurrence of not only heart failure and ischemic stroke but also cognitive decline. During episodes, symptoms such as palpitations negatively affect patients’ quality of life. Furthermore, AF is associated with high mortality rates and reduced healthy life expectancy; thus, its appropriate management is a crucial public health concern.^3^

Although radiofrequency catheter ablation (RFCA) has long been the standard treatment, it has become the first-line treatment in many centers. However, approximately 10% of PAF cases are driven by non–pulmonary vein (non-PV) triggers, which are increasingly recognized as an important contributor to postablation AF recurrence.^4^ Identifying non-PV AF before ablation may allow for the adoption of more individualized treatment strategies, such as the selection of appropriate catheters or ablation targets. However, currently, no noninvasive method has been established to detect the presence of non-PV triggers before the procedures.

Thus, in addition to well-known clinical risk factors such as aging, valvular heart disease, hypertension, diabetes, alcohol consumption, and sleep apnea, genetic predisposition is crucial in the development of AF.^5^

To date, genome-wide association studies (GWASs) have identified >150 genetic loci associated with AF.^6^ Among known variants, *PITX2* variants showed the strongest association with AF.^7^ *PITX2* was reported to suppress the development of automaticity-bearing cells in the left atrium and affected the embryological formation of the myocardial sleeves connecting the PVs to the left atrium.^8^ Minor alleles in *PITX2* variants may induce the ectopic expression of automaticity cells, which are normally confirmed to the sinoatrial node in the right atrium, within the left atrium, potentially acting as an AF trigger.^9^ Thus, although the molecular biological mechanism of AF has been elucidated, the pathways underlying non-PV triggers remain unclear.

For this study, we hypothesized that genetic factors may be associated with a non-PV focus of origin among individuals with AF. To test this hypothesis, we performed a comprehensive GWAS in a well-characterized cohort of patients with PAF who underwent RFCA, conducting the genetic analysis as a case–control comparison within the AF cohort, with patients with the non-PV trigger phenotype defined as cases and those with the PV-only phenotype defined as controls.

## Methods

### Participants

This retrospective study enrolled 1,091 consecutive patients with PAF who underwent initial RFCA at Hiroshima University Hospital between November 2009 and February 2022. The study protocol was approved by the Institutional Ethics Committee of the Graduate School of Biomedical and Health Sciences, Hiroshima University (E2002-9952) in accordance with the Declaration of Helsinki. All participants provided written informed consent.

### Genome-wide genotyping

The genetic analysis was performed as a comparison between patients with the non-PV trigger phenotype and those with the PV-only phenotype within an AF cohort.

The Asian Screening Array (Illumina) was used in the GWAS of all study participants. Genotype imputation was conducted using Minimac4 with a 5.6 K reference panel, which was constructed from phase 3 data of the 1000 Genomes Project and Japanese whole-genome sequencing data from the National Center for Geriatrics and Gerontology Biobank.^10^ The analysis included imputed variants with an imputation information score score ≥0.3.

The quality control (QC) criteria for individual samples were as follows: sample call rate ≥99%, genotype missingness <1%, correct sex specification, absence of close genetic relationships (PI_HAT >0.25 calculated using PLINK),^11^ and exclusion of population outliers from the cluster of East Asian populations based on principal component (PC) analysis using reference data from the phase 3 of the 1000 Genomes Project (https://www.internationalgenome.org). The following QC filters were applied to the genetic variants: genotyping call rate ≥99%, minor allele frequency >1%, and Hardy-Weinberg equilibrium *P* value >1 × 10^-6^ in controls. Common autosomal variants with consistent effect alleles that met all QC criteria were analyzed using logistic regression models that were adjusted for sex, age, and the top three PCs, which were calculated from the GWAS study samples, as implemented in PLINK. In addition to adjusting for the first three PCs (PC1–3) as covariates, sensitivity analyses were performed adjusting for PC1–5 and PC1–10. Furthermore, association analyses were also conducted using methods that accounted for the genomic relationship matrix, including generalized linear mixed model association tests (GMMAT) (https://github.com/hanchenphd/GMMAT) and Scalable and Accurate Implementation of GEneralized mixed model (SAIGE) (https://saigegit.github.io/SAIGE-doc/), in addition to the PLINK-based logistic regression analysis.

The association results were visualized using quantile-quantile and Manhattan plots, which were generated with the R package qqman (https://github.com/stephenturner/qqman). Additionally, lead single-nucleotide variants identified by the GWAS were visualized using LocusZoom (http://locuszoom.org),^12^ which produces regional association plots.

Moreover, allele frequencies in Japanese populations were obtained from the Tohoku Medical Megabank Organization database (54KJPN; https://jmorp.megabank.tohoku.ac.jp)^13^ and those in other populations from the Genome Aggregation Database (gnomAD) database (v4.1.0; https://gnomad.broadinstitute.org).^14^

### Echocardiography

In our institution, comprehensive transthoracic echocardiographic examinations were conducted before RFCA using commercially available ultrasound systems, such as the Vivid E9 (GE Healthcare) and the iE33 (Philips Medical Systems). To eliminate potential bias in the assessment, all echocardiographic studies were conducted and interpreted by experienced sonographers or cardiologists who were blinded to the findings of the GWAS.

### Electrophysiological study and RFCA

All antiarrhythmic drugs, except for amiodarone, were discontinued for at least five half-lives before RFCA. Amiodarone was discontinued at least 2 weeks before the procedure. Under selective PV angiography guidance, circular mapping catheters (Lasso; Biosense Webster) were placed in the superior and inferior left PVs, whereas a radiofrequency ablation catheter was inserted into the right superior PV. Other multipolar mapping catheters, such as PentaRay or Octaray (Biosense Webster), were also used as indicated. Additionally, four 5-Fr electrode catheters were introduced: a 10-pole catheter into the coronary sinus (CS) and three quadripolar catheters, each placed into the high right atrium (HRA), His bundle region, and right ventricle, with 5-mm spacing between each electrode.

To induce AF and identify its trigger sites, 10 μg of isoproterenol was intravenously administered via the right femoral vein before PV isolation (PVI). If AF was suspected to originate from the right inferior PV, mapping catheters were positioned in both the right superior and inferior PVs, and an additional 10 μg of isoproterenol was administered. If AF could not be induced, 10 mg of adenosine triphosphate was administered following isoproterenol administration.

A three-dimensional electroanatomical mapping system was employed: CARTO XP (Biosense Webster) from November 2009 to July 2011 and CARTO 3 (Biosense Webster) starting in August 2011. Both systems were used in conjunction with CARTOMERGE (Biosense Webster) for the integration of the computed tomography image.

To electrically isolate the left- and right-sided PVs, continuous circumferential PVI was conducted using an open-irrigated, 3.5-mm tip deflectable ablation catheter (THERMOCOOL SMARTTOUCH, Biosense Webster). In patients with non-PV AF triggers, targeted mapping and ablation were conducted to eliminate ectopic foci, which were defined as the earliest sites of spontaneous ectopic activity that initiates AF.

After PVI, an electrophysiological study (EPS) was conducted during a stable sinus rhythm. Baseline intracardiac electrograms were recorded, and the intra-atrial conduction time (HRA to His bundle electrogram), interatrial conduction time (HRA to the distal CS), atrial-to-His (interval, and His-to-ventricle interval were evaluated. The sinus node recovery time (SNRT) covered the time from the cessation of a 30-second HRA pacing burst to the return of the first spontaneous sinus beat. The corrected SNRT was calculated by subtracting the intrinsic sinus cycle length from the SNRT. To determine the maximal 1:1 conduction rate, atrioventricular nodal conduction was also assessed using incremental atrial pacing.

### Definition of AF origins

AF origins were classified into PV and non-PV groups according to the AF initiation site. AF originating from the posterior wall of the left atrium, including the PV antrum, was classified into the PV group because these structures share a common embryological origin.^15^ Non-PV triggers were defined as ectopic foci that initiated AF and reproducibly observed outside the PV antrum and posterior wall of the left atrium, such as the superior vena cava, crista terminalis, and CS. Patients who experienced AF recurrence following the initial PVI but did not undergo a repeat RFCA and those in whom PV reconnection could not be induced during the repeat RFCA and thus could not induce AF were excluded from the analysis.

### Follow-up after RFCA

Three months after the RFCA, antiarrhythmic and anticoagulant medications were continued. Specifically, antiarrhythmic medications were discontinued after 3 months, whereas anticoagulants were continued for patients with a CHADS2 score ≥2, based on Japanese guidelines (Japanese Circulation Society and the Japanese Heart Rhythm Society 2020 Guideline on Pharmacotherapy of Cardiac Arrhythmias). Patients underwent regular outpatient follow-up at 1, 3, and 6 months after the RFCA and every 6 months thereafter. At each visit, patients were assessed for AF recurrence by 12-lead electrocardiography and 24-hour Holter monitoring. Those who experienced palpitations or had an irregular pulse were advised to seek prompt evaluation at an outpatient clinic or emergency department. If arrhythmia was suspected, additional evaluations were conducted by 24-hour Holter monitoring, 14-day event recording, or portable electrocardiogram monitoring. Late AF recurrence was defined as an episode occurring >3 months after the RFCA, consisting of an episode lasting ≥30 seconds accompanied by palpitations or an episode lasting ≥30 seconds of AF, atrial flutter, or atrial tachycardia.

RFCA for AF was repeated in patients who experienced late AF recurrence and consented to the procedure. During the second session, the presence of PV reconnection was assessed using a multipolar mapping catheter, and as in the first session, non-PV focus induction was attempted as extensively as possible.

### Replication study

A replication study was conducted to validate the association of the genetic variant rs117203318, which was identified in the GWAS as significantly associated with AF originating from non-PV foci. The study population included a total of 371 consecutive patients with PAF who underwent initial RFCA at Hiroshima University Hospital between March 2022 and November 2024 and who provided informed consent for genetic analysis. Genotyping of rs117203318 was conducted by polymerase chain reaction (PCR)-based Invader assay (Third Wave Technologies) and QuantStudio 7 Flex Real-Time PCR System (Thermo Fisher Scientific)^16^ and the association of this variant with AF originating from non-PV foci was examined using Firth’s logistic regression adjusted for age and sex.

### Statistical analysis

Continuous variables are expressed as mean ± SD and categorical variables as count (percentage). Normality was assessed using the Shapiro-Wilk test. To compare baseline characteristics, echocardiographic parameters, and EPS parameters across groups, we used the chi-square test (or Fisher exact test where appropriate) for categorical variables and the Mann-Whitney *U* test for continuous variables. The AF recurrence rate after RFCA was evaluated using the Kaplan-Meier method, and between-group differences were evaluated using the log-rank test. A logistic regression analysis was conducted to identify predictors of AF recurrence. For the GWAS, logistic regression analysis was conducted to evaluate the association of each genetic variant with AF originating from non-PV foci, with adjustment for age, sex, and the top three PCs to account for population stratification. The association between rs117203318 variant and PCs was evaluated using Spearman’s correlation coefficient. Genome-wide significance was defined as *P* < 5.0 × 10^−8^. The linkage disequilibrium (LD) score intercept and slope were estimated with SE using LD score regression (https://github.com/bulik/ldsc) with East Asian reference LD panels. In the replication cohort, the association of the rs117203318 variant with AF originating from non-PV foci was assessed using Firth’s penalized logistic regression adjusted for age and sex under allelic and dominant genetic models. ORs with 95% CIs were calculated for each model. All statistical analyses were conducted using the JMP Pro Version 15.0.0 (SAS Institute), and Firth’s logistic regression was implemented using the R package “logistf” (https://cran.r-project.org/web/packages/logistf/index.html). A two-sided *P* value <0.05 was considered significant unless otherwise specified.

## Results

### Classification of AF origins

**Figure 1:**
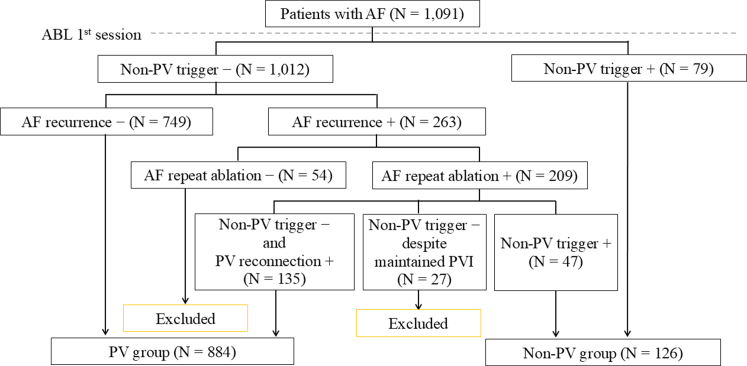
**Classification of Patients With Atrial Fibrillation Into the Pulmonary Vein and Non–Pulmonary Vein Groups Based on the Origin of Atrial Fibrillation Triggers** Of the 1,091 patients with atrial fibrillation who underwent initial catheter ablation, 54 with post-PV isolation atrial fibrillation recurrence without repeat radiofrequency catheter ablation and 27 with atrial fibrillation recurrence despite maintained PV isolation due to noninducibility were excluded, and 884 and 126 were classified into the pulmonary vein and non–pulmonary vein groups, respectively. ABL = ablation; AF = atrial fibrillation; non-PV = non–pulmonary vein; PV = pulmonary vein; PVI = PV isolation.

**Figure 2:**
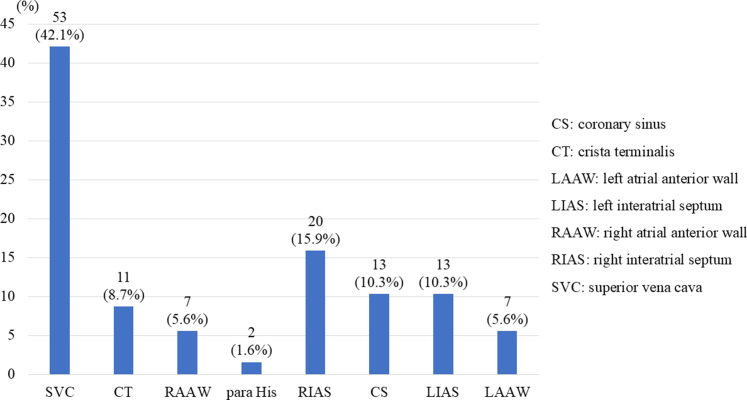
**Distribution of Non–Pulmonary Vein Atrial Fibrillation Trigger Sites** The superior vena cava was the most common source of atrial fibrillation among patients in the non–pulmonary vein group with identified trigger sites. CS = coronary sinus; CT = crista terminalis; LAAW = left atrial anterior wall; LIAS = left interatrial septum; RAAW = right atrial anterior wall; RIAS = right interatrial septum; SVC = superior vena cava.

Of the 1,091 patients with PAF who underwent initial catheter ablation (Figure 1), 54 who experienced AF recurrence following PVI but could not undergo a repeat RFCA were excluded from the analysis. Moreover, 27 patients without PV reconnection in whom AF could not be induced during the repeat RFCA were also excluded. Consequently, 884 and 126 patients were classified into the PV and non-PV groups, respectively. In the non-PV group, the superior vena cava was identified as the most frequent site of origin in those with a specific AF trigger site (Figure 2).

### Clinical characteristics, echocardiographic parameters, and electrophysiological study parameters

Table 1 summarizes the patient characteristics, echocardiographic parameters, and EPS parameters. The prevalence of hypertension was significantly lower in the non-PV group than in the PV group. No significant differences were found in the other baseline characteristics, such as age, sex, body mass index (BMI), history of diabetes mellitus, heart failure, and CHADS_2_ score between the 2 groups. Regarding echocardiographic findings, the ventricular septum was slightly thicker in the PV group than in the non-PV group; however, no significant differences were observed between the 2 groups in other echocardiographic parameters. However, regarding EPS parameters, the non-PV group showed significantly longer conduction times from the HRA to the His bundle electrogram and longer conduction times from the HRA to the distal CS than the PV group.

**Table 1:** Baseline Patient Characteristics, Echocardiographic Findings, and Electrophysiological Study Parameters

	All (N = 1,010)	PV Group (n = 884)	Non-PV Group (n = 126)	*P* Value
Age (y)	65.4 ± 10.8	65.5 ± 10.5	65.1 ± 12.3	0.84
Female (%)	332 (32.9)	282 (31.9)	50 (39.7)	0.082
BMI (kg/m^2^)	23.8 ± 3.5	23.9 ± 3.5	23.5 ± 3.5	0.25
AF duration (day)	941 ± 1,612	918 ± 1,532	1,106 ± 2097	0.20
Alcohol (%)	453 (44.9)	401 (45.4)	52 (41.3)	0.39
Hypertension (%)	624 (61.8)	561 (63.5)	63 (50.0)	0.004
Diabetes mellitus (%)	153 (15.2)	135 (15.3)	18 (14.3)	0.77
Stroke (%)	100 (9.9)	89 (10.7)	11 (8.7)	0.64
Structural heart disease (%)	80 (7.9)	69 (7.8)	11 (8.7)	0.72
Heart failure (%)	54 (5.4)	46 (5.2)	8 (6.4)	0.59
CHADs_2_ score	1.18 ± 1.0	1.19 ± 1.0	1.1 ± 0.96	0.39
Echocardiographic parameters				
LAVI (mL/m^2^)	37.6 ± 11.6	37.6 ± 11.4	37.4 ± 13.1	0.21
LAD (mm)	37.8 ± 5.8	37.9 ± 5.7	37.3 ± 6.5	0.12
LVDd (mm)	47.7 ± 4.6	47.7 ± 4.7	47.7 ± 4.1	0.81
LVDs (mm)	31.4 ± 4.2	31.4 ± 4.3	31.2 ± 3.7	0.68
IVS (mm)	8.8 ± 1.5	8.9 ± 1.5	8.6 ± 1.4	0.018
LVEF (%)	62.3 ± 5.6	62.2 ± 5.8	62.7 ± 4.6	0.58
E/e`	10.0 ± 3.9	10.1 ± 4.0	9.8 ± 3.5	0.36
EPS parameters				
Maximum SNRT (ms)	1,504 ± 586	1,502 ± 591	1,516 ± 543	0.91
CSRT (ms)	579 ± 514	577 ± 523	600 ± 438	0.56
1:1 AV nodal conduction (bpm)	140 ± 24	140 ± 24	138 ± 22	0.25
Conduction time				
HRA to HBE (ms)	35.0 ± 14.7	34.6 ± 14.6	37.9 ± 15.1	0.028
HRA to distal CS (ms)	94.6 ± 20.6	94.0 ± 20.2	99.2 ± 22.7	0.022
AH interval (ms)	102.9 ± 27.3	102.4 ± 27.7	107.1 ± 24.5	0.009
HV interval (ms)	42.1 ± 9.9	41.9 ± 9.9	43.3 ± 10.3	0.17

Values are mean ± SD or n (%).

AF = atrial fibrillation; AH = atrial-His; AV = atrioventricular; BMI = body mass index; CS = coronary sinus; CSRT = corrected sinus node recovery time; E/e` = mitral early diastolic peak to early diastolic mitral annular peak velocity; EPS = electrophysiological study; HBE = His bundle electrogram; HRA = high right atrium; HV = His-ventricular; IVS = interventricular septum; LAD = left atrial diameter; LAVI = left atrial volume index; LVDd = left ventricular end-diastolic diameter; LVDs = left ventricular end-systolic diameter; LVEF = left ventricular ejection fraction; non-PV = non–pulmonary vein; PV = pulmonary vein; SNRT = sinus node recovery time.

### AF recurrence rates

**Figure 3:**
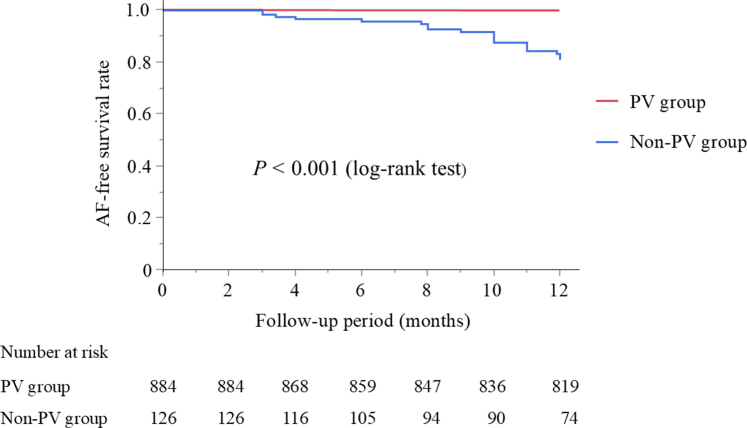
**Kaplan-Meier Analysis of Atrial Fibrillation Recurrence After the First Radiofrequency Catheter Ablation in the Pulmonary Vein and Non–Pulmonary Vein Groups** Over a mean follow-up of 20 ± 13 months, 139 of 884 patients (15.7%) in the pulmonary vein group and 71 of 126 (56.4%) in the non–pulmonary vein group had atrial fibrillation recurrence. The recurrence rate was significantly higher in the non–pulmonary vein group than in the pulmonary vein group (log-rank test, *P* < 0.001). AF = atrial fibrillation; PV = pulmonary vein.

Patients were monitored for AF recurrence for up to 60 months after the first RFCA. Kaplan-Meier curves for AF recurrence after the first RFCA are shown in Figure 3. During a mean follow-up period of 20 ± 13 months, AF recurred in 139 of 884 patients (15.7%) in the PV group and in 71 of 126 patients (56.4%) in the non-PV group. The recurrence rate was significantly higher in the non-PV group than in the PV group (log-rank test, *P* < 0.001), indicating that AF originating from non-PV foci contributes substantially to post-RFCA AF recurrence.

### GWAS results

A total of 7,012,601 variants from 1,010 patients, including 126 and 884 patients, fulfilled the QC criteria. There were no ancestry outliers in our GWAS cohort (Supplemental Figure 1), and scatter plots of PCs suggested substantial overlap between PV and non-PV individuals, with no clear separation indicating population stratification (Supplemental Figure 2).

**Figure 4:**
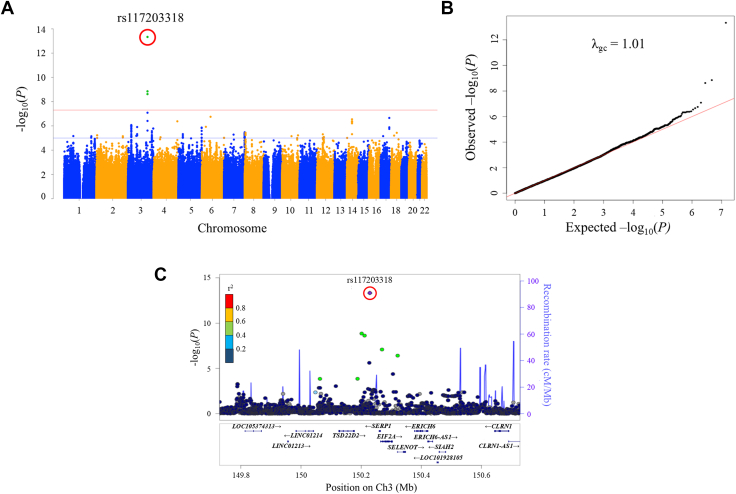
**Genome-Wide Association Study Analysis for Atrial Fibrillation Originating From Non–Pulmonary Vein Foci** A, Manhattan plot of the genome-wide association study result. A *P* value of 5 × 10^−6^ indicated by the blue line and a *P* value of 5 × 10^−8^ indicated by the red line. A genome-wide significant association was noted for the single-nucleotide variant rs117203318, located on chromosome 3 (*P* = 4.65 × 10^-14^). Its minor allele was significantly more common in patients with atrial fibrillation originating from non–pulmonary vein foci. B, Quantile-quantile plot of the genome-wide association study. Lambda GC = 1.01. C, The regional plot of rs117203318.

Figure 4 shows the results of the GWAS. The genomic inflation factor (λ_GC_) was 1.01 (Figure 4B). A genome-wide significant association was found for the single-nucleotide variant rs117203318 (*P* = 4.65 × 10^-14^; OR: 1.98; 95% CI: 1.49-2.62), located on chromosome 3. The result was largely consistent with adjusting for PC1-5, PC1-10, as well as in analyses performed using alternative GWAS methods that accounted for the genomic matrix, including GMMAT and SAIGE (Supplemental Figure 3). To assess whether rs117203318 reflects ancestry-related variation, we evaluated its correlation with PCs (PC1–10) derived from the study cohort; no significant correlations were observed (Supplemental Table 1). Although the LocusZoom plot (Figure 4C) appears to show relatively weak LD structure around the lead variant, this pattern is likely explained by the low-frequency and ancestry-specific nature of rs117203318, resulting in limited LD tagging by nearby variants. To further evaluate this possibility, we regressed single-nucleotide polymorphism–level association chi-square statistics on regional LD scores (defined as the sum of r^2^ within ±1 Mb using an East Asian reference panel) and found no positive correlation indicative of LD-driven inflation (Supplemental Figure 4). Consistently, LD score regression using East Asian reference panels yielded an intercept of 0.97 (SE = 0.006), indicating no evidence of population stratification. Its minor allele was significantly more commonly found in patients with AF originating from non-PV foci, indicating a strong association with AF originating from non-PV foci. The minor allele frequencies of rs117203318 were 5.4% overall in this study (non-PV group, 16.3%; PV group, 3.9%) and 6.7% in the Tohoku Medical Megabank Organization (ToMMo) 54KJPN database. The minor allele frequencies in other populations were 3.5% in East Asian, 2.5% in admixed Americans, 0.10% in African/African Americans, and 0.03% in non-Finnish Europeans (Table 2).

**Table 2:** Summary of Genome-Wide Association Study Results

Chr	Position (hg19)	A1	A2	Non-PV(A1A1/A1A2/A2A2)	PV(A1A1/A1A2/A2A2)	rsID	Nearest Gene	Annotation	OR (95% CI)	*P* Value
3	150228961	C	T	0/41/85	1/67/816	rs117203318	*SERP1*	Intergenic	1.98 (1.49-2.62)	4.65 × 10^-14^

A1 Allele Frequency	Non-PV	PV	Non-PV	PV	Overall in This Study (Non-PV + PV)	54KJPN	EAS∗	AFR∗	AMR∗	NFE∗
A1 allele count	42	69	16.3%	3.9%	5.4%	6.7%	3.5%	0.10%	2.5%	0.03%
A2 allele count	211	1,699								
Total allele count	252	1,768								

Values are genotype counts, allele counts, allele frequencies, ORs with 95% CIs, and *P* values.

54KJPN = Tohoku Medical Megabank Organization 54KJPN database; A1 = effect allele; A1A1 = individuals with a homozygous A1 genotype; A1A2 = individuals with a heterozygous genotype; A2 = noneffect allele; A2A2 = individuals with a homozygous A2 genotype; AFR = African/African American; AMR = admixed American; Chr = chromosome, EAS = East Asian; NFE = non-Finnish European; 54KJPN = Touhoku Medical Megabank Organization54KJPN database; other abbreviations as in Table 1.

∗ gnomAD v4.1.0.

### Univariable and multivariable analyses

In the univariable analysis, the absence of hypertension, conduction time from the HRA to the distal CS > 103 ms, and presence of minor alleles of rs117203318 were significantly associated with AF originating from non-PV foci. Although female sex was not significant in the univariable analysis, it emerged as an independent predictor in the multivariable logistic regression (OR: 1.61; 95% CI: 1.06-2.46; *P* = 0.027). The multivariable model further confirmed that the absence of hypertension (OR: 1.80; 95% CI: 1.20-2.71; *P* = 0.005), conduction time >103 ms (OR: 2.03; 95% CI: 1.35-3.05; *P* < 0.001), and rs117203318 minor alleles (OR: 5.82; 95% CI: 3.68-9.22; *P* < 0.001) remained significant independent predictors of AF originating from non-PV foci (Table 3).

**Table 3:** Univariable and Multivariable Analyses of Atrial Fibrillation Originating From Non-PV Foci

	PV Group (n = 884)	Non-PV Group (n = 126)	Univariate *P* Value	Multivariate	
				OR (95% CI)	*P* Value
Female (%)	282 (31.9)	50 (39.7)	0.082	1.61 (1.06-2.46)	0.027
BMI <23.8 kg/m^2^ (%)	466 (52.7)	72 (57.1)	0.35	1.09 (0.73-1.65)	0.67
Absence of hypertension (%)	323 (36.5)	63 (50.0)	0.004	1.80 (1.20-2.71)	0.005
E/e` >8.44 (%)	537 (60.8)	70 (55.6)	0.27	1.01 (1.00-1.02)	0.44
CSRT >550 ms (%)	336 (38.0)	47 (37.3)	0.88	1.13 (1.01-1.27)	0.84
Conduction time from HRA to distal CS > 103 ms (%)	274 (31.0)	58 (46.0)	<0.001	2.03 (1.35-3.05)	<0.001
Presence of minor alleles of rs117203318 (TC or CC) (%)	68 (7.7)	41 (32.5)	<0.001	5.82 (3.68-9.22)	<0.001

Values are mean ± SD or n (%), unless otherwise indicated.

Abbreviations as in Table 1.

### Replication study

Using the same criteria as in Figure 1, 309 and 37 patients were classified into the PV and non-PV groups, respectively. Meanwhile, 6 patients in whom the AF trigger could not be identified and 19 who did not undergo repeat RFCA despite AF recurrence were excluded. Patient characteristics, echocardiographic parameters, and EPS parameters of the replication study cohort are summarized in Supplemental Table 2. Genetic analysis of the rs117203318 was significantly associated with AF originating from non-PV foci (OR: 3.31; 95% CI: 1.40-7.55; *P* = 0.007) (Table 4).

**Table 4:** Association Between rs117203318 and Atrial Fibrillation Originating From Non-PV Foci in the Replication Study

	PV Group (n = 309)	Non-PV Group (n = 37)	OR (95% CI)	*P* Value
Age	69.3 ± 10.9	65.7 ± 12.9	0.97 (0.94-1.00)	0.051
Female (%)	118 (38.2)	18 (48.7)	1.97 (0.94-4.18)	0.071
Presence of effect allele of rs117203318 (%)	22 (7.1)	8 (21.6)	3.31 (1.40-7.55)	0.007
(A1A1/A1A2/A2A2)	0/22/287	1/7/29		

Values are mean ± SD or n (%), unless otherwise indicated.

Abbreviations as in Tables 1 and 2.

## Discussion

AF originating from non-PV foci is one of the major contributors to postablation AF recurrence.^17^ In the present study, the recurrence rate was significantly higher in the non-PV group than in the PV group. Several clinical predictors for AF originating from non-PV foci have been reported, such as female sex, low BMI, absence of hypertension, and high mitral early diastolic peak to early diastolic mitral annular peak velocity (E/e′) on echocardiography.^18^ More recently, a clinical risk score that incorporates female sex, sinus node dysfunction, previous AF ablation, and left atrial scar has been proposed.^19^ However, noninvasively predicting the presence of AF originating from non-PV foci during the initial ablation remains challenging. In the present cohort, AF originating from non-PV foci was significantly more common in patients without hypertension, and multivariable analysis further identified female sex as an independent predictor. These findings are consistent with previous reports. However, no significant associations were observed with BMI, E/e′, or sinus node dysfunction.

In our genetic analysis, a genome-wide significant association was identified between AF originating from non-PV foci and the single-nucleotide variant rs117203318 located on chromosome 3q25.1. Previous GWAS have identified loci near *PITX2* as strong genetic determinants of AF susceptibility when comparing AF cases with non-AF controls.^20^ However, no genome-wide significant association was observed at the *PITX2* locus in the present study. This difference may be explained by the study design. The present analysis compared 2 phenotypic subgroups within an AF cohort (non-PV trigger vs PV-only trigger), rather than AF cases vs non-AF controls. Therefore, loci that broadly influence AF susceptibility, such as *PITX2*, may not necessarily distinguish these subgroups. Instead, variants related to atrial substrate characteristics that predispose to non-PV triggers may emerge.

The variant identified in our study lies closest to *SERP1* and is located near several genes, including *TSC22D2*, *EIF2A*, and *SELENOT*, some of which are expressed in cardiac tissue and are involved in cellular stress responses and atrial remodeling.^21^^,^^22^

*SERP1*, an endoplasmic reticulum (ER) stress-associated membrane protein, stabilizes newly synthesized proteins under ER stress.^23^ In AF, impaired ER homeostasis due to intrinsic and extrinsic factors, such as inflammation, oxidative stress, and ischemia, induces ER stress, which promotes atrial remodeling and contributes to AF development and persistence.^24^ Experimental studies have demonstrated that pharmacologically reducing ER stress via chemical chaperones or inhibiting the phosphorylation of eukaryotic initiation factor 2α suppresses electrical remodeling (eg, action potential shortening and calcium current reduction), thereby attenuating AF progression in canine and Drosophila models.^25^ Thus, the rs117203318 variant may impair *SERP1* expression or function, potentially affecting the cellular response to ER stress and promoting atrial myocyte injury and electrophysiological alterations. Previous studies have suggested that atrial electrical remodeling and structural changes may be associated with non–PV foci.^26^ In this context, the *SERP1*-related pathway may contribute to the development of an atrial substrate that facilitates AF initiation from ectopic activity arising outside the PVs into AF.

*TSC22D2* encodes a leucine zipper transcription regulator. Its protein family, including *TSC22D1*, respond to cardiac overload by facilitating fibroblast activation and collagen synthesis,^27^^,^^28^ potentially contributing to atrial fibrosis and development of an arrhythmogenic substrate. Although the cardiac function of *EIF2A* has not been fully elucidated, it was reported to act as a translation initiation factor under stress conditions.^29^ *SELENOT* is implicated in maintaining ER homeostasis and defending against oxidative stress and has been reported to exert cardioprotective effects on ischemia-reperfusion models.^30^ Furthermore, the selenoprotein family was suggested to play protective roles against oxidative stress and cardiovascular diseases.^31^ Selenoprotein dysfunction may mediate enhanced oxidation of the cardiac ryanodine receptor 2 and abnormal Ca^2+^ leakage, potentially promoting ectopic activity.^32^ Taken together, these findings propose that the minor alleles of rs117203318, located near genes implicated in myocardial fibrosis and cellular stress responses, may increase the risk of AF originating from non-PV foci by disrupting the transcriptional and translational regulation of key pathways involved in atrial remodeling.

Moreover, this study revealed that intra-atrial conduction times were significantly longer in the non-PV group than in the PV group. Another study demonstrated that reduced intra-atrial conduction velocity was an independent predictor of post-PVI AF recurrence.^33^ Intra-atrial conduction delay may reflect an increased arrhythmogenic substrate sustaining AF, thereby increasing the risk of AF originating from non-PV foci and contributing to AF recurrence. However, the rs117203318 variant was not associated with intra-atrial conduction times (Supplemental Table 3).

### Study limitations

This study has several limitations. First, non-PV foci were identified based on induction testing, potentially overlooking noninducible triggers. Second, the GWAS and replication analyses were conducted in a Japanese cohort from a single institution. Although replication was conducted in an independent cohort, data were still derived from the same institution, which may limit the generalizability of the findings. In addition, this was a single-center study, and a truly independent external validation cohort was not available. Therefore, the present findings should be considered hypothesis-generating and require confirmation in independent cohorts with standardized phenotyping for non-PV triggers. Third, because the present GWAS compared AF patients with non-PV triggers and those with PV-only triggers, the identified locus may reflect genetic influences on electrophysiological phenotypes captured by the testing protocol, such as trigger inducibility or response to pharmacologic provocation, rather than general AF susceptibility. Consistent with this possibility, rs117203318 has not been reported as an AF susceptibility locus in previous large-scale GWAS including East Asian populations, such as the multiancestry meta-analysis by Roselli et al.^34^ Therefore, it remains uncertain whether this locus contributes directly to the pathogenesis of non-PV triggered AF. Fourth, the minor allele frequency of rs117203318 was relatively higher in Japanese and East Asian populations than in African American and non-Finnish European populations. This observation suggests that rs117203318 may represent an ethnicity-specific variant enriched in East Asian populations. In addition, comorbidity profiles differ substantially across ethnic groups; in particular, obesity and heart failure are more prevalent in North American populations.^35^ Therefore, differences in underlying clinical and environmental backgrounds should also be taken into consideration when interpreting these findings. To validate our findings, future multicenter studies including diverse ethnic groups are warranted.

## Conclusions

AF originating from non-PV foci remains a major cause of recurrence even in patients with PAF; however, its prediction remains challenging. In this study, rs117203318, a novel genetic variant, was identified, which was significantly associated with AF originating from non-PV foci. Genes near this locus may be involved in atrial remodeling and cellular stress responses, presenting a novel pathophysiological pathway underlying the occurrence of non-PV triggers (Central Illustration).

Perspectives **COMPETENCY IN MEDICAL KNOWLEDGE:** Non–pulmonary vein (non-PV) triggers represent an important mechanism of atrial fibrillation (AF) recurrence after ablation. The minor allele of rs117203318 and prolonged intra-atrial conduction may identify patients predisposed to AF with non-PV foci.

**TRANSLATIONAL OUTLOOK:** Future studies are needed to clarify the biological mechanisms linking rs117203318 to atrial remodeling and to determine whether integrating genetic and electrophysiological markers can guide individualized ablation strategies.

**Central Illustration:**
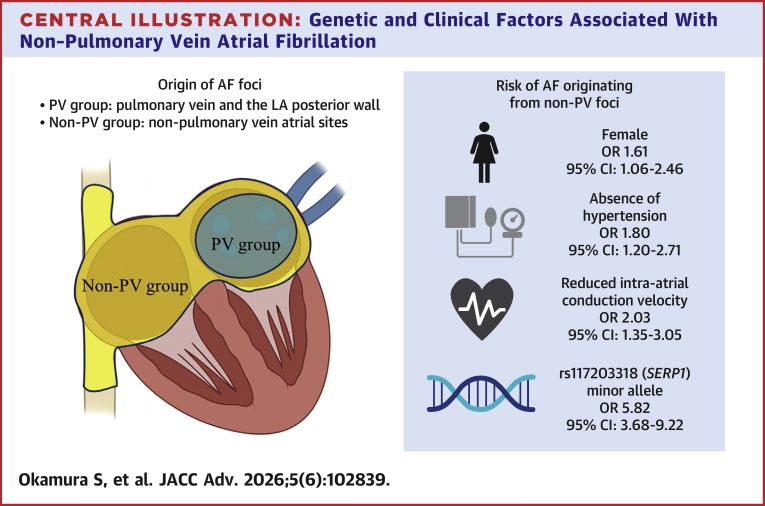
**Genetic and Clinical Factors Associated With Non–Pulmonary Vein Atrial Fibrillation** Non–pulmonary vein foci were defined as atrial triggers excluding the pulmonary veins and left atrial posterior wall. The minor allele of rs117203318 near *SERP1* was significantly associated with atrial fibrillation originating from non–pulmonary vein foci. Female sex, absence of hypertension, and reduced intra-atrial conduction velocity were also associated with increased susceptibility to non–pulmonary vein atrial fibrillation. AF = atrial fibrillation; non-PV = non–pulmonary vein; PV = pulmonary vein.

## Funding support and author disclosures

Dr Nakano was supported by The 10.13039/501100000646Japan Society for the Promotion of Science (JSPS KAKENHI; grant number: 17K09501). All other authors have reported that they have no relationships relevant to the contents of this paper to disclose.
