# Supplementary material for: Genetic Variants Associated With Nonpulmonary Vein Triggers of Atrial Fibrillation: A Genome-Wide Association Study
Source: JACC Adv. 2026 May 29;5(6):102839. doi: 10.1016/j.jacadv.2026.102839 (PMC13308242; doi:10.1016/j.jacadv.2026.102839)

Supplemental Table 1. Correlation between rs117203318 and PCs

SNP	PC	Spearman rho	*P*
rs117203318	PC1	0.021	0.51
	PC2	0.037	0.24
	PC3	-0.039	0.21
	PC4	0.031	0.33
	PC5	-0.014	0.66
	PC6	0.016	0.61
	PC7	0.043	0.17
	PC8	0.046	0.15
	PC9	0.022	0.49
	PC10	0.034	0.27

PC, principal component

Supplemental Table 2. Baseline characteristics of patients with PAF and comparison between the PV and non-PV groups in the replication study

	All (n = 346)	PV group (n = 309)	Non-PV group (n = 37)	*P* value
Age (years)	68.9 ± 11.2	69.3 ± 10.9	65.7 ± 12.9	0.10
Female (%)	136 (39.3)	118 (38.2)	18 (48.7)	0.22
BMI (kg/m^2)	23.9 ± 3.6	24.0 ± 3.6	23.1 ± 3.6	0.077
Alcohol (%)	139 (40.2)	128 (41.4)	11 (29.7)	0.17
Hypertension (%)	208 (60.1)	191 (61.8)	17 (46.0)	0.063
Diabetes mellitus (%)	62 (17.9)	59 (19.1)	3 (8.1)	0.10
Stroke (%)	44 (12.7)	39 (12.6)	5 (13.5)	0.88
Structural heart disease (%)	50 (14.5)	46 (14.9)	4 (10.8)	0.51
Heart failure (%)	51 (14.7)	45 (14.6)	6 (16.2)	0.78
CHADs2 score	1.5 ± 1.3	1.6 ± 1.2	1.2 ± 1.3	0.048
Echocardiographic parameters				
LAVI (mL/m^2)	37.3 ± 14.8	37.5 ± 15.3	35.7 ± 9.6	0.73
LAD (mm)	38.1 ± 6.4	38.4 ± 6.1	36.2 ± 8.1	0.18
LVDd (mm)	47.7 ± 6.6	47.7 ± 6.2	47.7 ± 4.9	0.88
LVDs (mm)	31.9 ± 5.9	32.0 ± 6.1	31.7 ± 4.4	0.97
IVS (mm)	8.8 ± 1.6	8.8 ± 1.6	8.6 ± 1.7	0.32
LVEF (%)	61.5 ± 8.1	61.3 ± 8.4	62.6 ± 5.9	0.67
E/e`	11.3 ± 5.7	11.4 ± 5.6	10.6 ± 6.9	0.080
EPS parameters				
Maximum SNRT (ms)	1743 ± 634	1747 ± 633	1717 ± 651	0.82
CSRT (ms)	688 ± 575	681 ± 575	751 ± 582	0.37
1:1 AV nodal conduction (bpm)	128 ± 25	127 ± 25	134 ± 26	0.34
Conduction time				
HRA to HBE (ms)	45.1 ± 16.0	44.9 ± 15.8	47.0 ± 17.6	0.66
HRA to distal CS (ms)	106.3 ± 20.5	106.0 ± 20.7	109.6 ± 18.4	0.31
AH interval (ms)	110.8 ± 29.7	110.8 ± 29.4	110.8 ± 32.2	0.86
HV interval (ms)	44.0 ± 10.1	43.9 ± 10.0	44.1 ± 10.4	0.61

AH, atrial-His; AV, atrioventricular; BMI, body mass index; bpm, beats per minute; CS, coronary sinus; CSRT, corrected sinus node recovery time; E/e`, mitral early diastolic peak to early diastolic mitral annular peak velocity; EPS, electrophysiological study; HBE, His bundle electrogram; HRA, high right atrium; HV, His-ventricular; IVS, interventricular septum; LAD, left atrial diameter; LAVI, left atrial volume index; LVDd, left

ventricular end-diastolic diameter; LVDs, left ventricular end-systolic diameter; LVEF, left ventricular ejection fraction; mm, millimeter; ms, millisecond; PAF, paroxysmal atrial fibrillation; PV, pulmonary vein; SNRT, sinus node recovery time

Supplemental Table 3. Baseline characteristics and intra-atrial conduction times according to rs117203318 genotypes (dominant model)

	rs117203318 (T>C)		*P* value
	TT (n = 901)	TC + CC (n = 109)	
Age (years)	65.4 ± 10.8	65.4 ± 10.5	0.96
Female (%)	299 (33.2)	33 (30.3)	0.54
BMI (kg/m^2)	23.8 ± 3.5	23.9 ± 3.5	0.76
Hypertension (%)	559 (62.0)	65 (61.8)	0.63
Conduction time			
HRA to HBE (ms)	35.0 ± 14.7	34.8 ± 15.0	0.87
HRA to distal CS (ms)	94.3 ± 20.6	96.8 ± 20.7	0.24
AH interval (ms)	103.2 ± 27.1	100.7 ± 29.8	0.38
HV interval (ms)	42.0 ± 10.1	42.6 ± 8.2	0.53

AH, atrial-His; BMI, body mass index; CS, coronary sinus; HBE, His bundle electrogram; HRA, high right atrium; HV, His-ventricular; ms, millisecond

Supplementary Figure 1. Principal component analysis of the GWAS study samples.

Study participants were plotted together with reference populations from the 1000 Genomes Project. The samples cluster tightly with East Asian populations, indicating genetic homogeneity of the cohort.

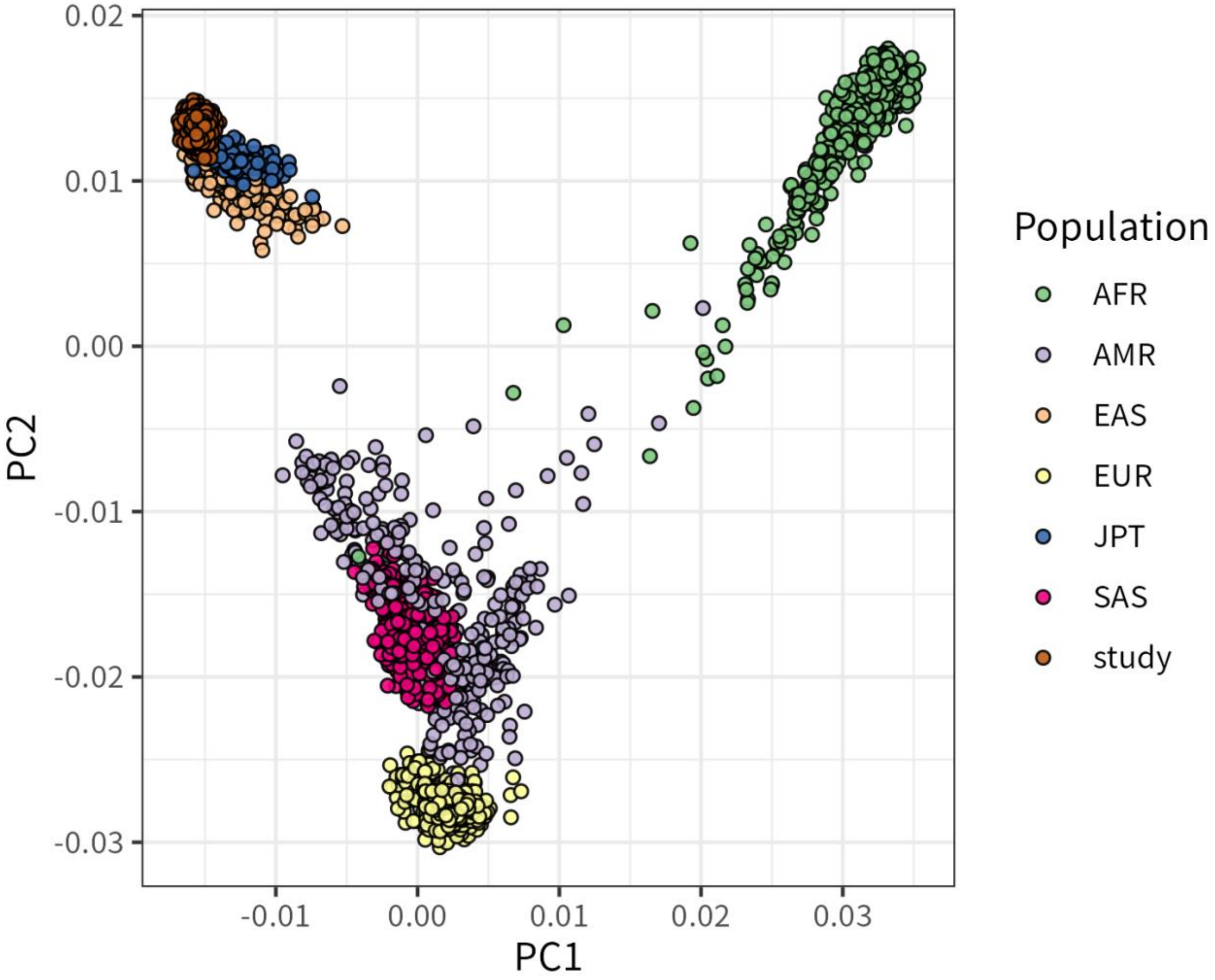

Supplementary Figure 2. Principal component analysis plots of PV and non-PV individuals.

Scatter plots of PC1 vs PC2 and PC2 vs PC3 show substantial overlap between the PV and non-PV groups, with no evidence of systematic separation indicating population stratification.

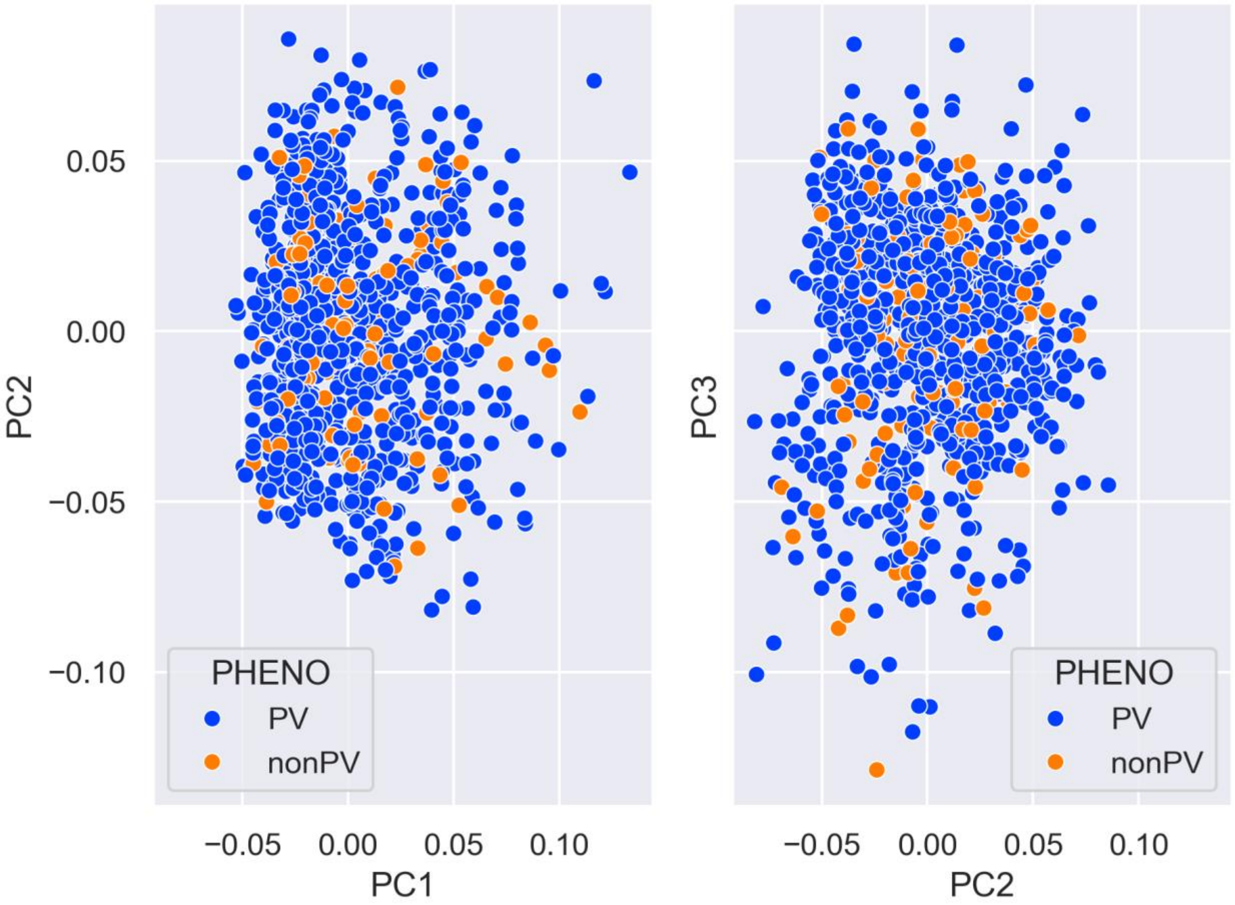

Supplementary Figure 3. The results of sensitivity analyses

(A) The GWAS results adjusting for age, sex, and PC1–5

(B) The GWAS results adjusting for age, sex, and PC1–10

(C) The GWAS result using GMMAT

(D) The GWAS result using SAIGE.

A.

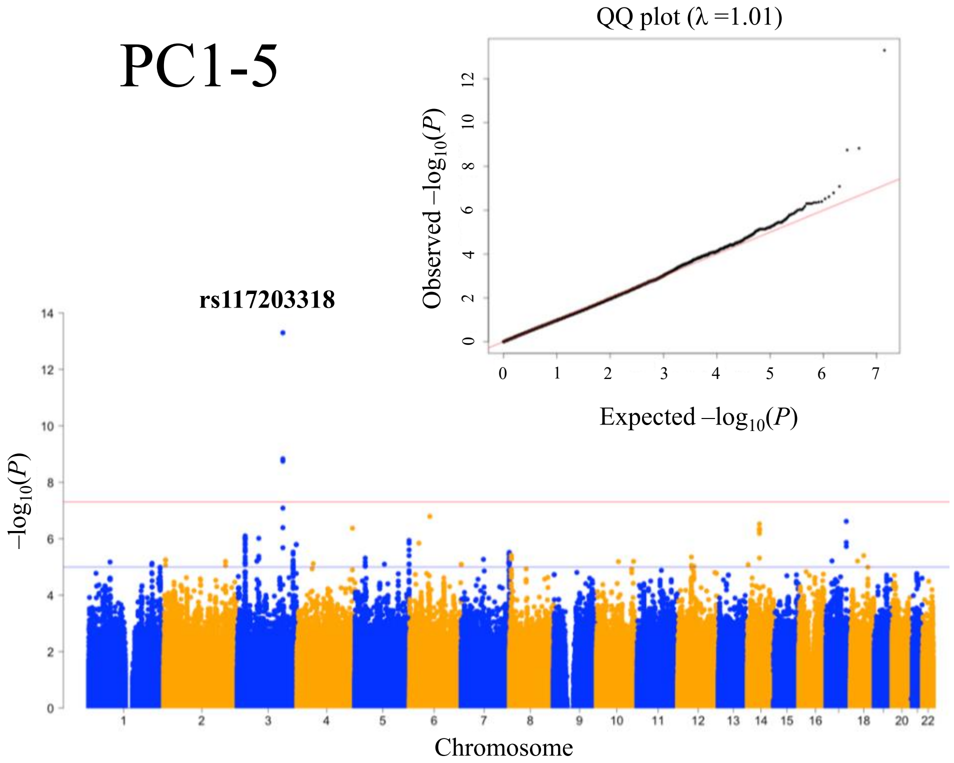

B.

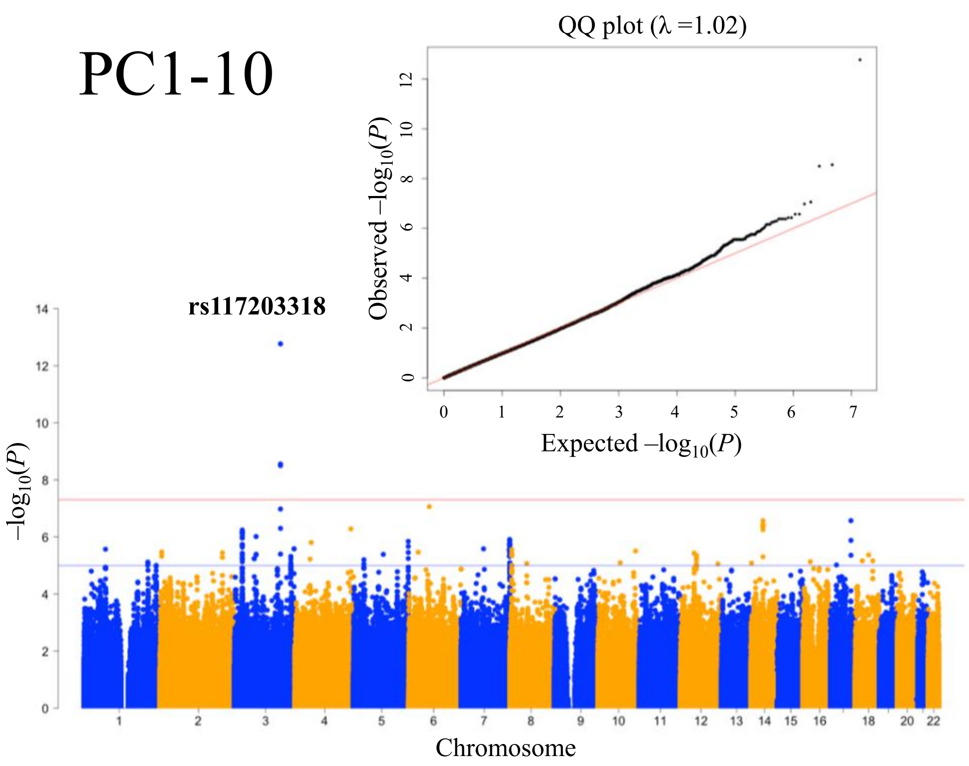
PC1-10
QQ plot (λ =1.02)
Observed –log10(P)
Expected –log10(P)
rs117203318
–log10(P)
Chromosome

C.

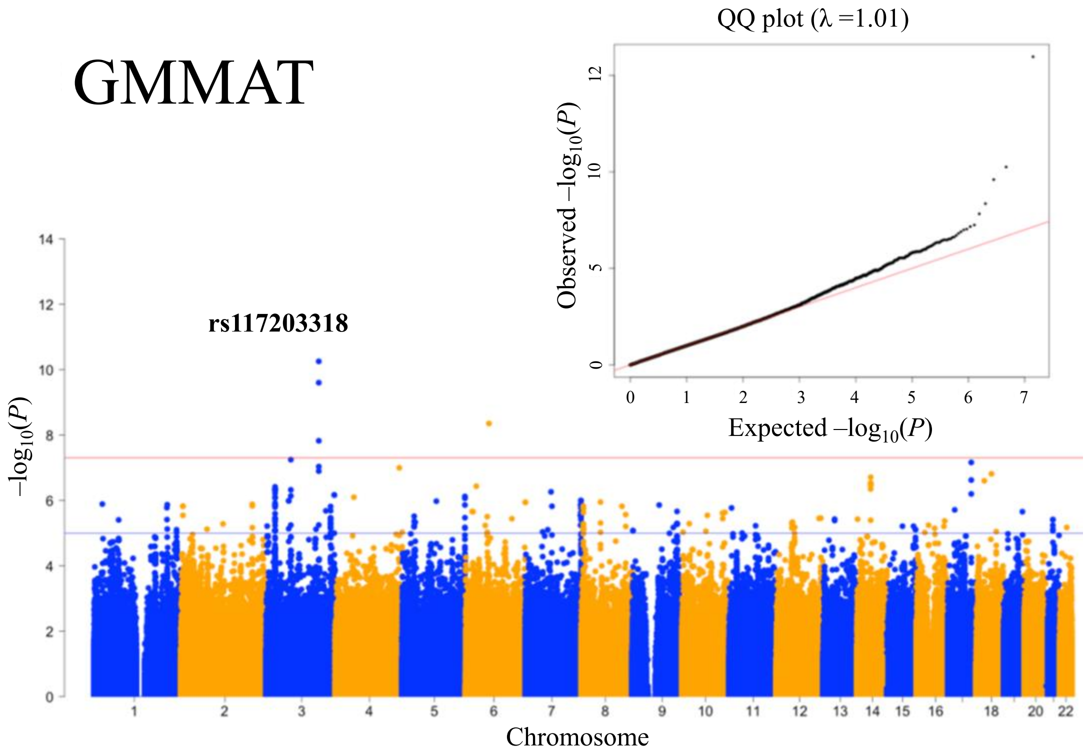
GMMAT
QQ plot (λ =1.01)
Observed –log10(P)
Expected –log10(P)
rs117203318
–log10(P)
Chromosome

D.

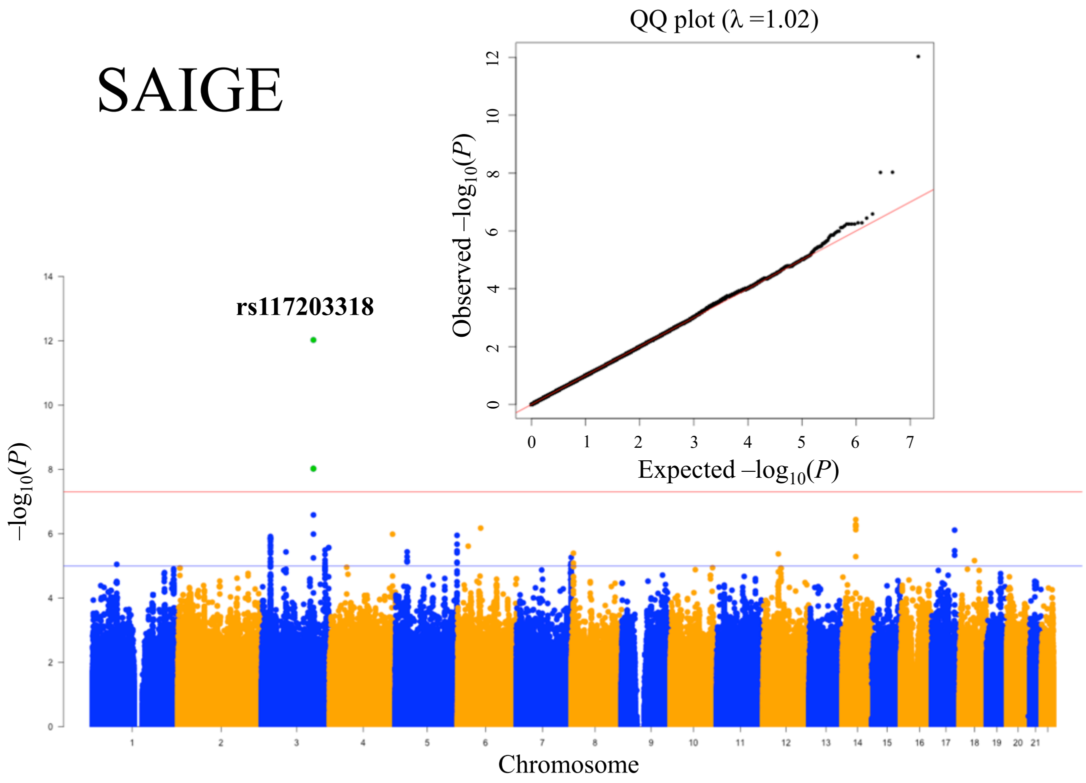
SAIGE
QQ plot (λ =1.02)
Observed –log10(P)
Expected –log10(P)
rs117203318
–log10(P)
Chromosome

Supplementary Figure 4. Association χ² statistics vs regional LD scores around rs117203318.

Regression of SNP-level association χ² statistics on regional LD scores around rs117203318 (defined as the sum of r² within ±1 Mb using an East Asian reference panel). No positive correlation was observed, suggesting that the association signal is unlikely to be driven by linkage disequilibrium–related inflation.

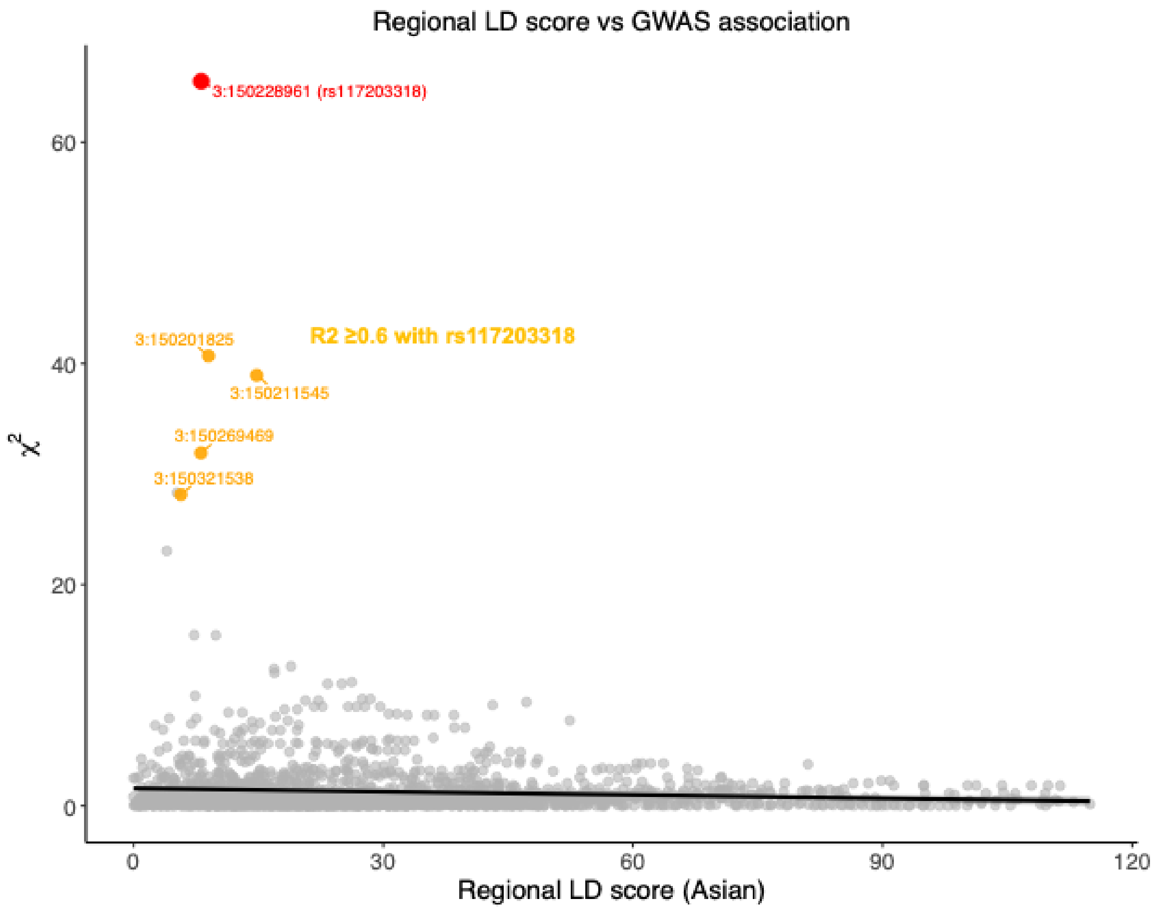

Supplement: Supplemental_Material [file mmc1.pdf]
